# Type 2 Diabetes Mellitus in Saudi Arabia: Prevalence, Risk Factors, and Management Strategies: A Review

**DOI:** 10.2174/0118715303361062250122100238

**Published:** 2025-04-03

**Authors:** Raed Aldahash, Khaled K. Aldossari, Naji Aljohanni, Fahad Alsabaan, Wael Alzahrani, Abdullah Alwabel, Ahmad M. N. Alhendi

**Affiliations:** 1 Department of Medicine and Endocrinology, Ministry of National Guard Health Affairs, King Abdullah International Medical Research Center and King Saud Bin Abdulaziz for Health Science, Riyadh, Kingdom of Saudi Arabia;; 2 Department of Family and Community Medicine, College of Medicine, Prince Sattam Bin Abdulaziz University, Al-Kharj, Kingdom of Saudi Arabia;; 3 Obesity, Endocrine and Metabolism Center, King Fahad Medical City, Riyadh 12231, Kingdom of Saudi Arabia;; 4 Department of Endocrinology, College of Medicine, Alfaisal University, Riyadh 11533, Kingdom of Saudi Arabia;; 5 Department of Endocrinology, Security Forces Hospital, Riyadh, Kingdom of Saudi Arabia;; 6 Department of Endocrinology and Diabetes, Prince Sultan Military Medical City, Riyadh, Kingdom of Saudi Arabia;; 7 Department of Medicine Riyadh, King Saud Medical City, Central Region, 12372, Kingdom of Saudi Arabia;; 8 Department of Medical Affairs, Hikma Pharmaceuticals, P.O. BOX 106229, Riyadh 11666, Kingdom of Saudi Arabia

**Keywords:** Diabetes mellitus, obesity, HbA1c, SGLT2i, dapagliflozin, hypertension

## Abstract

Diabetes mellitus (DM) is a major health problem and a leading cause of death in the Kingdom of Saudi Arabia (KSA). The World Health Organization (WHO) ranks KSA as the seventh country with the highest diabetes prevalence in the world. The healthcare and treatment costs for diabetes have risen by more than 500% in the last two decades. Obesity is the main risk factor for type 2 diabetes mellitus (T2DM), which involves insulin resistance and β-cell dysfunction. Genetic and environmental factors also influence the development of T2DM. There are various options for controlling blood glucose in T2DM patients, including a new class of oral drugs called sodium-glucose transport protein 2 inhibitors (SGLT2i). These drugs reduce glucose reabsorption and increase glucose excretion in the kidney. They can be used at any stage of diabetes and have benefits such as lowering blood pressure, A1C levels, and body weight. Dapagliflozin is one of the SGLT2 inhibitors that T2DM patients well tolerate. This review examines the impact of T2DM in KSA, its risk factors and complications, and the role of Dapagliflozin in its management. It also provides expert opinions on the current situation of T2DM in KSA.

## 1 INTRODUCTION

Diabetes is derived from a Greek term that signifies a siphon (dia = through + bainein = to go) [1]. It is a chronic, non-infectious condition that develops when the pancreas is unable to produce enough insulin or when the body is unable to use the insulin that is produced [2].

DM, according to the WHO and the International Diabetes Federation (IDF), is the biggest worldwide health issue of the twenty-first century [3]. It is also the fourth most prevalent chronic disease identified after hypertension, arthritis, and dyslipidemia. Diabetes is the leading cause of macrovascular and microvascular problems, creating a massive global health burden [4]. Diabetes-related complications and mortality can create social and economic problems that have a significant negative impact on people's lives as well as the livelihoods of their families, enterprises, and entire societies [3]. The reasons for this global epidemic of diabetes include population growth, aging, affluence, sedentary lifestyles, and poor eating choices eating choices, all of which contribute to the serious problems of obesity and physical inactivity [2].

**Fig. (1):**
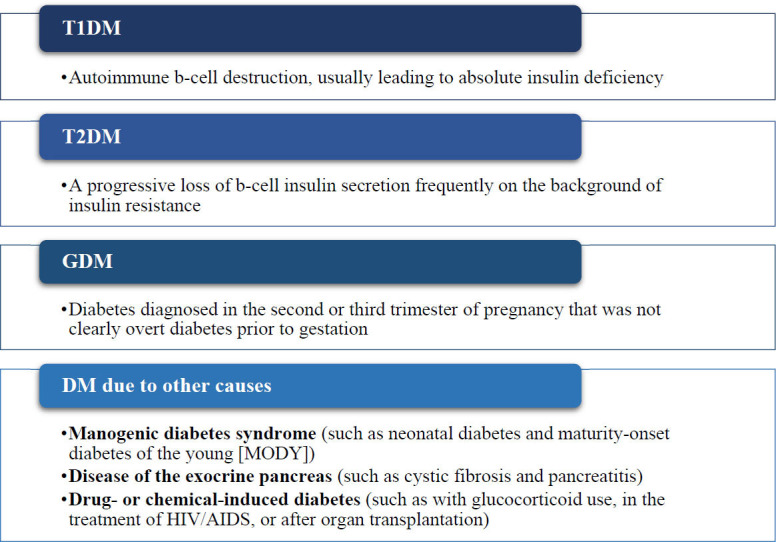
Classification of DM.

The National Diabetes Data Group (NDDG) and the WHO have defined four major types of diabetes: Type 1 Diabetes Mellitus (T1DM), T2DM, Gestational Diabetes Mellitus (GDM), and diabetes secondary to other conditions (Monogenic diabetes syndrome; Diseases of the exocrine pancreas; Drug- or chemical-induced diabetes) (Fig. **1**) [1, 5, 6].

### 1.1 Diabetes Burden (Prevalence, Incidence, and Demography)

The burden that DM places on society is demonstrated by both the disease's exponential population rise and the rising frequency of early deaths caused by it [2]. In 2009, the diabetic population, including both T1DM and T2DM, was approximately 285 million individuals. Subsequently, this figure increased to 366 million in 2011, 382 million in 2013, 415 million in 2015, 425 million in 2017, 463 million in 2019, and 537 million in 2021. IDF projections indicate a further rise to 643 million by 2030, with an anticipated peak of 783 million by 2045 (46% increase) [7, 8]. Several studies have predicted an increasing rise in DM, especially in emerging nations [6]. New estimates of the prevalence of DM, mortality attributed to DM, and healthcare costs associated with diabetes place a significant strain on the global, social, financial, and health systems. Furthermore, it is estimated that 374 million people have Impaired Glucose Tolerance (IGT) and about 21.3 million postpartum women who deliver live babies will experience some degree of hyperglycemia during pregnancy. Diabetic complications caused 5 million deaths globally in 2017 among people aged 20 to 99.

T2DM accounts for about 90% of all instances of diabetes. One in eleven persons worldwide is said to be affected by it, and more than 80% of people with T2DM live in low- to middle-income nations [9, 10].

### 1.2 Circumstances in Arab Countries

According to the most recent IDF statistics, there is a growing trend of DM incidence and prevalence in the Arab area, which appears to be greater than the worldwide average of DM rise [2]. In the Middle East and North Africa (MENA) region, as reported by the IDF (10th ed. 2021), diabetes affected over 73 million individuals (1 in 6 adults) aged between 20 and 79 in 2019. Projections for 2030 suggest an anticipated increase of 95 million people with diabetes (PwD) in the MENA region, and by 2045, this figure is expected to rise to 136 million (87% increase) [7]. As of now, the Arab governments have not placed sufficient emphasis on the high incidence of non-communicable illnesses such as DM, especially when it comes to addressing the policy variations across nations and generally subpar execution. Among Arab-speaking countries, Kuwait, Lebanon, Qatar, Saudi Arabia, Bahrain, and the United Arab Emirates (UAE) are six of the 10 countries that have the highest prevalence rates of diabetes. Furthermore, according to the information available, there are 20 Arab nations where roughly 20.5 million individuals have diabetes, and another 13.7 million have IGT or pre-diabetes [2]. Data from the Gulf revealed high prevalence rates of T2DM. The reported prevalence rates were 25.7%, 16.1%, and 21% in Bahrain, Oman, and Kuwait, respectively [9].

Around 170,000 adult deaths in the Arab world are linked to diabetes, accounting for more than 10% of all fatalities there. The consequences of diabetes include blindness, amputations, renal failure, and cardiovascular disorders (CVDs), all of which can result in temporary and permanent disability [11]. End-stage Renal Disease (ESRD) was allegedly mostly caused by diabetes in Jordan (29.2%), but the rate in the UAE was 23.3%. Since 30% of PwD develop kidney disease, diabetic kidney disease is likely another significant health issue in the Arab world [2, 12, 13].

### 1.3 The Situation in the Kingdom of Saudi Arabia (KSA)

KSA is not excluded from this global epidemic, and diabetes is the most challenging health problem facing this country [14]. According to the WHO, KSA ranks second in the Middle East region and seventh around the globe in terms of diabetes prevalence [2]. In 1992, there were about 0.9 million cases of diabetes, but by 2010, there were 2.5 million cases, or a 2.7-fold rise in incidence rates in less than two decades, according to research from the Saudi Arabian Ministry of Health (MoH). The temporal factor's coefficient showed that the prevalence rate rose after the year 1992 [10].

Jarrar *et al.* (2023), in a systematic review, found that the pooled prevalence of T2DM in KSA between 2000 to 2020 was 16.4% [15]. Alwadeai *et al.* (2023), in a meta-analysis, revealed that the prevalence of T2DM among the general adult population in KSA was 28% [16].

According to the 10th edition of the IDF (2021), around 4.27 million of the 24.19 million Saudi citizens between the ages of 20 and 79 have diabetes (prevalence 17.7%). Additionally, it is estimated that there are nearly 1.3 million undiagnosed diabetic persons in the country, including approximately 27,884 children and adolescents [7, 17]. The IDF Atlas also revealed that by 2035, there might be 7.5 million people in KSA who will be suffering from T2DM [7].

A large proportion of PwD in the KSA who are older than 30 years are unaware of their condition, accounting for 40.3% of the total. Moreover, as 25.5% of the Saudi population above 30 years have pre-diabetes and 28.7% are obese, the country is likely to see a significant increase in the number of people with T2DM [18].

Additionally, to estimate the current and future burden of T2DM in KSA, Al-Quwaidhi *et al.* (2014) developed the ‘Saudi IMPACT Diabetes Forecast Model’ in 2013, using a validated Markov model that incorporated the trends of obesity and smoking as the main risk factors [19]. They also compared their model predictions with those from other sources, such as the IDF Atlas and the Global Burden of Disease (GBD) project. They found that their model estimates were consistent with the 2005 national survey and very similar to the GBD estimates. A study in 2000 by the GBD project estimated the prevalence of diabetes in men at 17.5% and women at 17.7%, respectively. However, a later model developed specifically for Saudi Arabia, the Saudi IMPACT Diabetes Prediction Model, predicted slightly different figures: 17.7% for men and 16.4% for women. The IDF predictions of the overall diabetes prevalence were far lower than those of this study, coming in at 16.7% in 2011 and 20.8% in 2030, as opposed to 29.2% in 2011 and 44.1% in 2022 [19].

Meo SA *et al.* (2016) performed a thorough analytic investigation in 2016 to highlight the incidence and prognosis of T2DM in the KSA. This systematic review included 21 papers in total, and it regressed the 33-year prevalence rate of diabetes (1982-2015) against the time to calculate the projected prevalence of T2DM based on the findings of the research that was accessible. In KSA, the prevalence of T2DM is 32.8%. Nevertheless, the prevalence is anticipated to be 40.37% in 2025 and 45.36% in 2030 [20]. IDF predicts that approximately one-quarter of Saudi adults will have diabetes by 2045 [21].

### 1.4 Risk Factors for DM in the KSA Population

There are two categories of risk factors associated with T2DM: modifiable and non-modifiable risk factors.

#### 1.4.1 Modifiable Risk Factors

##### 1.4.1.1 Lifestyle Patterns and Urbanization

KSA noticed an insidious increase in the prevalence and incidence of DM soon after the rapid industrialization, which resulted in a remarkable rise in the standard of living and the adoption of a more ‘Westernized’ lifestyle [2, 22]. Diabetes has increased to over 25% of the adult population as a result of poor eating habits and a decline in physical activity levels across the country. One study found that diabetes prevalence was considerably greater in urban regions, even among low- or middle-income groups, indicating that both urbanization and monthly income had an impact on diabetes [2]. According to Al-Nozha *et al.,* a community-based national epidemiological health study discovered that 25.5% of Saudis who live in urban areas have diabetes, compared to 19.5% of Saudis who live in rural regions. Regional variations are also observed, with the Southern area (18.2%) supporting a more rural lifestyle and population with a lower inclination towards obesity than the Northern and Eastern provinces (27.9% and 26.4%, respectively), which exhibit higher prevalence rates of DM [2, 23].

##### 1.4.1.2 Dietary Pattern

KSA's distinct nutritional patterns may contribute to the prevalence of diabetes in the population. Common food choices include sweets, rice, dates, and meat dishes which is high in fat and carbohydrates, as well as popular items such as French fries, kabsa (mixed rice dish), and baked goods. Studies suggest these dietary choices could be significant determinants of diabetes risk in KSA [2].

##### 1.4.1.3 Physical Activity

Numerous studies support the notion that decreasing activity levels are linked to the global epidemic of DM. Research on the physical activity profile of the Saudi population shows that most Saudis are not physically active enough to benefit from exercise on a long-term basis [2]. The consequences of physical inactivity on the health of Saudi society are addressed in a quick assessment of the published statistics by Al-Hazzaa *et al.* (2004) about the level of physical activity in the KSA population. In that assessment, it was noted that Saudi children and adults' overall inactivity rates ranged from 43.3% to 99.5%. The middle-aged years saw the highest percentage of inactivity (30-49 years) [2, 24].

##### 1.4.1.4 Obesity

The risk of developing T2DM in KSA is significantly increased by obesity and overweight, both of which are correlated with lifestyle [3]. Obese and overweight people have a much higher chance of developing diabetes than those with an ideal weight. Compared to those with an ideal weight (BMI 18.5-24.9 kg/m^2^), obese individuals (≥30 kg/m^2^) have a seven-fold higher risk, and overweight individuals (25-29.9 kg/m^2^) have a three-fold higher risk. This risk steadily increases with increasing BMI. However, even within the normal BMI range, the risk is not zero. For example, a study in the NHS found that women with a BMI of 23.0-24.9 kg/m^2^ had a 2.67 times greater risk of developing diabetes compared to women with a lower BMI [25]. The majority of the increase in the incidence of DM has been seen because of lifestyle changes that have resulted in more sedentary, high-fat, and obese lifestyles. Both characteristics, which are simple to prevent and may be reversed, are viewed as normal in KSA due to their high occurrence. According to Rasheed P.'s research on obesity in the Eastern Province of Saudi Arabia, it has reached epidemic proportions among women, especially those over 40. This age group had 78.4% overweight females compared to the preceding age groups, where up to 30-40% of the female population was overweight or obese [26].

#### 1.4.2 Non-Modifiable Risk Factors

##### 1.4.2.1 Gender Differences

Diabetes was shown to increase substantially with age in both sexes. Nonetheless, worldwide data indicated a modest gender disparity in the diabetic population in 2013 or 2035 [2]. According to the IDF 2021 atlas, women aged 20 to 79 years are predicted to have a slightly lower prevalence of diabetes than men (10.2% *vs.* 10.8%) worldwide. Male PwD outnumber female PwD by 17.7 million in 2021 [7].

Diabetes is more common in women than in men in KSA, with 42% and 37.2%, respectively. High intakes of fat, free sugars, salt, and cholesterol have become more prevalent in the Saudi Arabian diet due to the Westernization of the country's cuisine [22]. Al-Nozha *et al.* reported a lower level of leisure time physical activity among the Saudis (6.1% in men and 1.9% in women) [23, 24]. The drastic changes in lifestyle, together with Saudis' inherited propensity for diabetes and the high rate of consanguineous marriages, have been linked to an increase in T2DM prevalence over the same period [27, 28].

##### 1.4.2.2 Age Differences

Diabetes prevalence is on the rise, particularly with age. According to forecasts for 2021, young adults (20-24 years) have the lowest prevalence at 2.2%. This number jumps significantly with age, reaching 24.0% for those aged 75-79 in 2021, and is expected to climb further to 24.7% by 2045.

This trend holds in Saudi Arabia as well. Studies have shown that age is the strongest risk factor for diabetes, with individuals over 60 being particularly vulnerable to diabetes-related complications. A study by Al-Rubeaan (SAUDI-DM study) found that diabetes risk increases with age, with the highest prevalence (44.7%) occurring in those 65 and older compared to younger age groups (14.7% in 30-44-year-olds and 37.5% in 45-65-year-olds). In fact, the prevalence increases by roughly 10% for every 10 years of age. According to the study, the greatest non-modifiable risk factor for diabetes in the KSA cohort was the age between 45 and 64. This was discovered to be more significant than the outcomes noted for other ethnic groups [29].

##### 1.4.2.3 Genetic Factors

Genetics play a strong role in diabetes risk: Having a family member with DM significantly increases the risk for other relatives. This risk is particularly high for first-degree relatives. Family history is a valuable tool used in population-based screening programs to identify individuals at higher risk. Studies have shown a perfect concordance rate (100%) for DM among monozygotic twins, suggesting a strong genetic component. Additionally, around 25% of individuals diagnosed with DM report a family history of the disease. This highlights the significance of inherited factors in DM development and emphasizes the importance of considering family history when assessing an individual's risk [2, 30, 31].

In addition to the Saudi population's inherited propensity for diabetes, consanguineous marriages are very common [28]. According to a survey, first-degree relatives make up the most consanguineous individuals (22.4%), accounting for the 56% of all congenialities. Consanguineous marriages were considerably more common in rural areas (59.5%) than in cities (54.7%). Another study revealed a consanguinity percentage of 57.7%, with first-cousin weddings accounting for 28.4% of all unions and marriages to distant relatives accounting for 14.6% [32].

### 1.5 Genetic Markers or Environmental Factors Affecting Diabetes in Saudi Arabia

Fifty Single Nucleotide Polymorphisms (SNPs) that incur a higher risk of T2DM has been investigated in the Saudi population. However, only 12 SNPs were successfully replicated in the Saudi population as risk factors for type 2 diabetes. The Rs7903146 variant near transcription factor 7-like 2 (TCF7L2) showed the highest level of statistical significance among all the detected variants in the Saudi population (1.13x10^-8^) [33].

The TCF7L2 was replicated by association studies in the European, Asian, and African ethnicities and was found to have the strongest effect on the type 2 diabetes risk. Juxtaposed with another zinc finger (JAZF1), have been reported to affect the development of pancreatic beta cells, which are responsible for insulin production. The TCF7L2, Wolfram syndrome 1 gene (WFS1), and hepatocyte nuclear factor 4 alpha (HNF4A) affect insulin processing and maturation in the pancreatic beta cells. Dual specificity phosphatase 9 (DUSP9) has been suggested to affect insulin signaling and stimulation [33].

The ATP-binding cassette transporters A1 (ABCA1), cyclin-dependent kinase inhibitor 2B (CDKN2A/B), and potassium voltage-gated channel (KCNQ1)are associated with the secretion of insulin from the pancreatic cells. While all other detected variants affect the maturation of beta cells and signaling and production of insulin, insulin receptor substrate 1 (IRS-1) are associated with modulating insulin action in target cells [33]. Exposure of Saudis to elevated levels of physical inactivity, higher consumption of fast foods, and lower consumption of vegetables and fruits are important explanations for the increased prevalence of metabolic syndrome (MS) that increases the risk of diabetes in the country. Additionally, the reported lower levels of physical activities make an important contribution to the increased prevalence of diabetes among Saudi females compared with Saudi males [33].

### 1.6 Economic Burden of Diabetes

Diabetes places a significant financial burden on nations, healthcare systems, individuals with diabetes, and their families.^7^ Healthcare expenses for diabetic patients are typically over 10 times higher ($3686 *vs.* $380) than for people without diabetes [9]. For persons aged 20 to 79, the expected worldwide healthcare costs for those with diabetes were USD 850 billion in 2017 and USD 966 billion in 2021. The IDF predicts that between 2030 and 2045, the overall cost of diabetes-related medical expenses will be USD 1.03 trillion and USD 1.05 trillion, respectively [7].

### 1.7 Economic Costs of Diabetes in Saudi Arabia

Diabetes-specific healthcare spending in Saudi Arabia indicates the additional costs the country is paying to address just one illness. In 2010, the MoH spent more than $9.4 billion on healthcare, of which $0.9 billion went on treating diabetes, or $1 out of every $11 spent on healthcare [2].

The Saudi MoH spent an estimated 17 billion Saudi Riyals (SAR) in 2014 on the direct care of type 1, type 2, and gestational diabetes for Saudi nationals only. According to the same study, the direct expenses of type 1, type 2, and gestational diabetes management in the total Saudi population, which includes both Saudi nationals and ex-pats, are estimated to be SAR 25 billion, with the amount predicted to climb to SAR 39.8 billion in the future [34]. Over the past 18 years, diabetes patients have spent more than 500% of their income on healthcare [2].

A research-based study conducted by Alhowaish *et al.* revealed that people diagnosed with diabetes, on average, have medical healthcare expenditures that are ten times higher ($3,686 *vs.* $380) than what expenditures would be in the absence of diabetes. Over 96% of all medical healthcare expenditures attributed to diabetes are incurred by persons of Saudi nationality, with the remaining 4% incurred by persons of non-Saudi nationality. The population aged 45-60 incurs 45% of diabetes-attributed costs, with the remaining population under age 15 incurs 3.8%, age 15-44 incurs 27.5%, and age 60 and above incurs 23.8%.

The actual national healthcare burden due to diabetes is likely to exceed the $0.87 billion estimated in this study because it omits the indirect costs associated with diabetes, such as absenteeism, lost productivity from disease-related absenteeism, unemployment from disease-related disability, and lost productivity due to early mortality by disease [35].

A short-term decision-analytic model with a 1-year time horizon was developed from a payer's perspective in the United States setting. The objective of study was to compare 1-year costs and benefits of dapagliflozin (DAPA), a sodium-glucose cotransporter-2 (SGLT-2) inhibitor, with those of other treatments for type 2 diabetes (T2D), such as glucagon-like peptide-1 receptor agonists (GLP-1RAs), sulfonylureas (SUs), thiazolidinediones (TZDs), and dipeptidyl peptidase-4 inhibitors (DPP-4i), all combined with metformin. Data for costs and quality-adjusted life-years (QALYs) associated with a per-unit change in these clinical endpoints were taken from published literature. The total annual medical cost for DAPA was less than that for GLP-1RA ($186 less), DPP-4i ($1,142 less), SU ($2,474 less), and TZD ($1,640 less). Treatment with DAPA resulted in an average QALY gain of 0.0107, 0.0587, 0.1137, and 0.0715 per treated patient when compared with GLP-1RA, DPP-4i, SU, and TZD, respectively. Incremental cost-effectiveness ratios (ICERs) for DAPA *vs* SU and TZD were $19,005 and $25,835, respectively. DAPA was a cost-saving option when compared with GLP-1RAs and DPP-4is. This analysis showed that DAPA was cost-saving compared with GLP-1RA and DPP-4i, and cost-effective compared with SU and TZD in the US setting over 1 year [36].

### 1.8 Health-Related Quality of Life (HRQoL)

Diabetes may have a significant impact on a patient's physical and mental health by causing both short-term and long-term difficulties, physical symptoms, and lifestyle modifications, as well as helplessness and emotional anguish. Many factors have been demonstrated to have an impact on diabetes patients' HRQoL. They include a person's age, gender, and body mass index (BMI), as well as blood sugar, glycated hemoglobin (HbA1c), illness duration, obesity, dyslipidemia, hypertension, and a history of heart disease [37].

Studies from KSA, other Middle Eastern countries, and the rest of the world show that diabetes impairs the QoL of patients, but the level of impairment was not the same across the studies [38].

Al-Shehri *et al.,* found that patients older than 50 had lower HRQoL than those younger than 50. The longer duration of diabetes was substantially associated with poor HRQoL, and those in lower socioeconomic strata reported having diabetic complications. The study also discovered that gender, education level, economic situation, and comorbidities from diabetes mellitus were independent risk factors for HRQOL, with females having poorer HRQOL than males [37]. A review by Robert *et al.* (2017) found that KSA’s direct spending on diabetes was close to 14% of the overall health expenditure, and the study advised increasing patient health and HRQoL to lower the societal and private costs of diabetes treatment in KSA. Better socioeconomic status and good control of CV risk factors were associated with higher HRQoL among patients with diabetes [2].

### 1.9 Type-II Diabetes Related Comorbidities

Individuals with T2DM are more likely to develop cardiovascular problems, ESRD, and hypertension because they have a higher risk of risk factors that are comparable to them, such as obesity, endothelial dysfunction, vascular inflammation, and dyslipidemia. However, it has also been discovered that those with T2DM are more likely to have depression, thyroid disorders, and chronic obstructive pulmonary disease (COPD) [39]. Undiagnosed or poorly managed T2DM can cause microvascular consequences, such as bouts of hypoglycemia and hyperglycemia, lower limb amputation, blindness, and kidney failure [40]. DM can also aggravate major infectious diseases such as TB, HIV/AIDS, and malaria [2].

A recent study from the KSA city of Al Ahsa found that individuals with diabetes had a high incidence of chronic problems. The high rates of hypertension, dyslipidemia, and obesity are a few of the important comorbidity factors. Overall, 72.72% of the research subjects had one or more problems related to diabetes mellitus. 33.39% of them only experienced one problem, 25.29% experienced two issues, and 15% experienced three or more. The study also showed that overall; complications were much more common in women than in men [41].

A recent study using data from the Saudi National Diabetes Registry revealed that the prevalence of diabetic nephropathy in the KSA was 10.8% overall, with distinct subtypes of 1.2% microalbuminuria, 8.1% macroalbuminuria, and 1.5% ESRD. Furthermore, it was noted that age and length of the patient's diabetes course were significant risk factors that had a significant impact on the prevalence of diabetic nephropathy. These risk factors ranged from 3.7% in patients in the 25-44 age group and a duration of >5 years to 21.8% in patients under the age of 65 and a diabetes course of ≥15 years [42].

### 1.10 Measurable Strategies

It is essential to test asymptomatic adults and adolescents for prediabetes and asymptomatic T2DM due to the rising prevalence of diabetes in KSA. According to the American Diabetes Association (ADA), all people 45 and older should have a T2DM screening performed by a healthcare professional every three years. Nevertheless, the recommendations of other significant health organizations diverge somewhat from those of the ADA. For instance, starting at age 30, the American Society of Clinical Endocrinologists advises high-risk individuals to have yearly screenings. The 30-39 age range has a significant prevalence of diabetes, according to earlier research conducted in KSA. Moreover, to lower the prevalence of diabetes, it is advised that every Saudi male and female over the age of 30 undergo screening for T2DM and pre-diabetes [2].

#### 1.10.1 Screening of T2DM in Asymptomatic Adolescents

Starting at age 10 or puberty (whichever comes first), children with a BMI exceeding the 85th percentile (overweight) or the 95th percentile (obese) and who have additional risk factors listed in Fig. (**2**) should undergo risk-based screening for prediabetes and/or asymptomatic T2DM [1].

#### 1.10.2 Screening of T2DM in Asymptomatic Adults

**Fig. (3):**
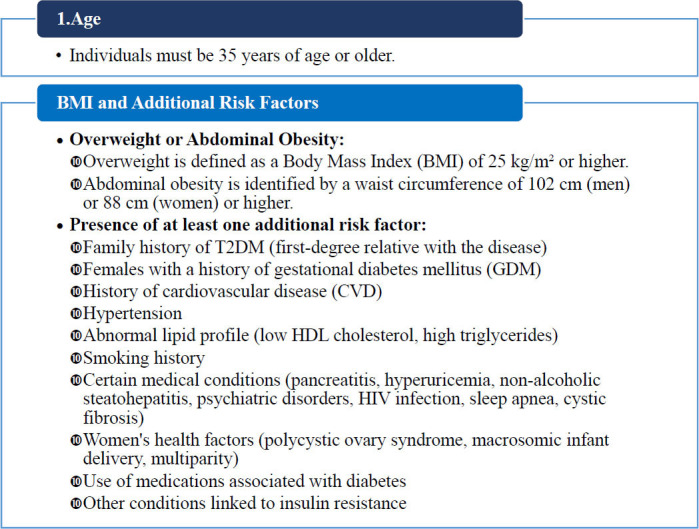
Screening approach for T2DM in asymptomatic adults.

The increasing prevalence of T2DM among adults aged ≥35 in KSA is a cause for concern. This trend, coupled with rising obesity rates and increasingly sedentary lifestyles, necessitates proactive measures to identify and manage the disease at an early stage. Recognizing the importance of early detection, the Saudi National Diabetes Center (SNDC) recommends a screening approach for all asymptomatic adults (Fig. **3**) [1].

### 1.11 Criteria for the Screening and Diagnosis of DM

DM diagnosis can be established through various plasma glucose criteria. These include fasting plasma glucose (FPG) or the 2-hour plasma glucose (2-h PG) levels obtained following a 75-g oral glucose tolerance test (OGTT). Another criterion for diagnosis is the A1C standard, which reflects average blood glucose levels over the past 2-3 months. Additionally, random blood sugar (RBG) levels are considered in the diagnostic process. Each of these criteria offers distinct insights into an individual's glucose metabolism, contributing to a comprehensive assessment for the accurate identification and diagnosis of diabetes Table **1** [1].

### 1.12 Ambulatory Glucose Profile (AGP)

The AGP is an internationally agreed upon and defined framework for processing, plotting, and analyzing data obtained by detecting tissue or blood glucose levels in terms of treatment-relevant parameters. It gives PwD a thorough snapshot of their glucose profile and streamlines clinical decision-making for physicians and the diabetes team. Due to the data's strong visual representation, PwD may also better grasp their glycemic management. While examining the AGP, it is also crucial to consider each patient's unique daily or weekly glucose profiles since they help describe the patterns seen there more precisely. The causes of individual hyperglycemic and hypoglycemic events and trends can, therefore be analyzed and discussed with the person with diabetes [43]. The AGP reports making it possible to apply tried-and-true customized care for diabetic patients in daily life [44].

#### 1.12.1 Patient Profiling

After DM diagnostic confirmation, a thorough medical assessment of the patient (in the initial visit and follow-up visit) is advised to help classify individuals into subtypes for a better allocation of treatment. Comorbidities (autoimmune diseases, cancer, pancreatic adenocarcinoma, non-alcoholic fatty liver disease, hypogonadism, HIV, anxiety disorders, depression, *etc.*) and the likelihood of DM complications (diabetic retinopathy, nephropathy, neuropathy, stroke, heart disease, peripheral vascular disease, or medication associated hypoglycemia) are assessed as part of this [1].

#### 1.12.2 Goal Setting

**Fig. (4):**
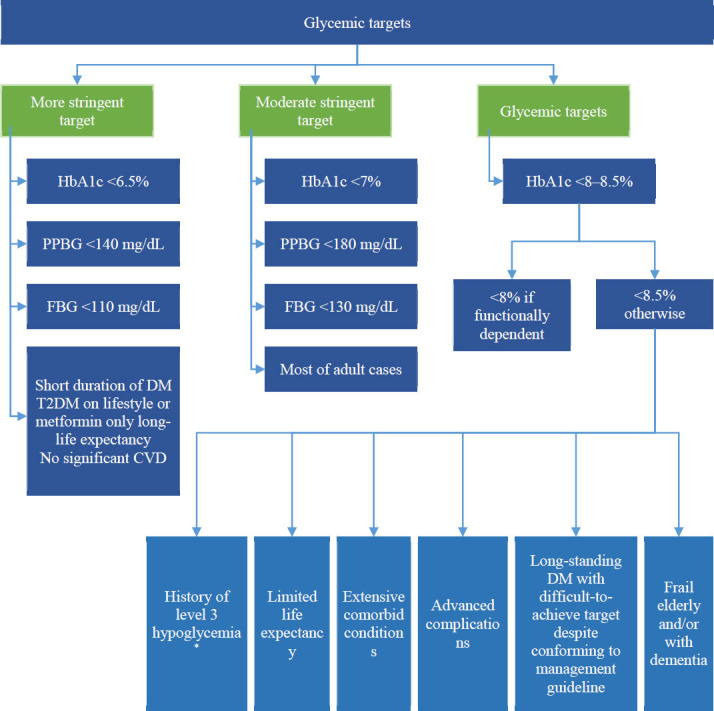
Glycemic targets. **Abbreviations:** HbA1c: Glycated haemoglobin; PPBG: Postprandial blood glucose; FBG: Fasting blood glucose; DM: Diabetes mellitus; CVD: Cardiovascular disease. *Altered mental and/or physical functioning. Requires assistance from another person for recovery.

The HbA1c goal in most persons with DM is <7%. Both the Diabetes Control and Complications Trial (DCCT) and the United Kingdom Prospective Diabetes Study (UKPDS) revealed that tight glycemic control, HbA1c <7% (53 mmol/mol), demonstrated a decrease in microvascular problems and probably macrovascular consequences [45]. In certain other situations, the objective may be between 6.5% and 8%. Within a range of around 6.5% to 8%, the appropriate goal is selected based on the circumstances (Fig. **4**) [1].

### 1.13 Treatment Paradigm of Type-II Diabetes in Saudi Arabia

**Fig. (5):**
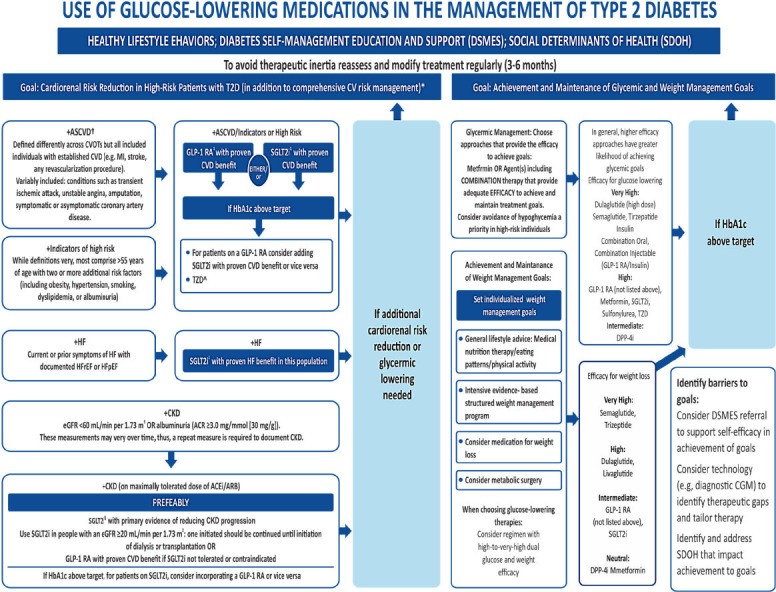
Pharmacologic treatment for the management of glycemia in T2DM. The integrated care of type 2 diabetes must consider the person with diabetes as an individual concerning specific preferences and values, social determinants of health, barriers to care, comorbid conditions, degree of hyperglycemia, risks of complications, and susceptibility to medication side effects. The overall goal of managing type 2 diabetes is maintaining quality of life and avoiding complications. The management approach must be holistic and multifactorial and account for the lifelong nature of type 2 diabetes. The choice of glucose-lowering agents should be directed by the individual profile of the person with type 2 diabetes, in particular the presence of comorbidities, risk of side effects, preferences, and context as per the above figure.

Patients with T2DM are treated with education, assessments for micro- and macrovascular problems, efforts to attain near normoglycemia, reduction of cardiovascular and other long-term risk factors, and avoidance of medications that might worsen insulin or lipid metabolic abnormalities. Based on individual considerations like age, life expectancy, and comorbidities, each of these treatments and objectives must be modified. Improved insulin sensitivity, increased insulin availability, delayed delivery and absorption of carbohydrates from the gastrointestinal tract, increased urinary glucose excretion, or a combination of these strategies are some of the mechanisms by which treatments improve glycemic control. Body weight management should be taken into consideration as a treatment goal in addition to glycemia for patients who are overweight, obese, or who have a pattern of distribution of adipose tissue that is metabolically unfavorable [46]. To make well-informed decisions on the pharmacologic management of glycemia in T2DM, (Fig. **5**) provides information about the pharmacologic control of glycemia in T2DM patients [47].

First-line therapy depends on comorbidities, patient-centered treatment factors, and management needs and generally includes metformin and comprehensive lifestyle modification. Other medications (Biguanides, Thiazolidinediones (TZD), Sulphonyl-ureas, Dipeptidyl Peptidase IV (DPP IV) inhibitors (DPP-4 inhibitors), Glucagon-Like Peptide 1 receptor agonists (GLP-1RA), SGLT2i, with or without metformin based on glycemic needs, are appropriate initial therapy for individuals with T2DM. GLP-1RA, TZD, and SGLT2i in T2DM individuals with or at high risk of atherosclerotic cardiovascular disease (ASCVD). SGLT2i in T2DM individuals with Heart Failure (HF). SGLT2i and GLP-1RA in T2DM individuals with Chronic Kidney Disease (CKD) [47].

Moreover, to ascertain the treatment patterns among T2DM patients (n = 455 adults) in KSA, Levy A.R. *et al.* (2014) undertook research. Throughout the trial, the number of changes (drug replacement/ removal/addition) and the frequency of usage of treatment regimens were calculated. The most popular regimens were oral combination therapy (41%) and insulin + oral combination therapy (32%), according to the study findings. Overall, 44% of research participants received some form of insulin treatment. At the end of the research, 49% already had insulin therapy. During the study, T2DM individuals experienced an average of 1.3 therapy shifts, with minimal variance in T2DM duration [48].

### 1.14 SGLT2i: Benefits Beyond Glycemic Control

The main goal of the mechanism of traditional antihyperglycemic medications is to reduce blood glucose levels by affecting insulin production, insulin resistance, beta cell activity, or carbohydrate metabolism. The unique mechanism of action of drugs in the SGLT2i family is unrelated to insulin resistance and beta cell activity. Since these medications have insulin-independent glucose-lowering ability, they can be administered at any stage of diabetes. These medications not only address the fundamental antihyperglycemic function but also the metabolic aspects of diabetes, such as hypertension, dyslipidemia, and obesity. They are, therefore, the medication of choice for people with these comorbid disorders [49].

Additionally, by lowering the renal threshold for glucose excretion, SGLT-2 inhibitors suppress renal glucose reabsorption and thereby increase urinary glucose excretion (UGE). Hyperglycemia is thus ameliorated. However, SGLT-2 inhibitors inhibit reabsorption of only ~ 30-50% of the glucose filtered by the kidney. One hypothesis is that SGLT-2 inhibitors may be actively secreted into the PCT such that the amount of SGLT-2 inhibitor in the PCT is limited by saturation of renal secretion of the inhibitor at high doses, and, depending on the site of secretion, the inhibitors may be unable to act on upstream SGLT-2. Another hypothesis is that SGLTs other than SGLT-2 may play a greater role in glucose reabsorption than is currently believed. SGLT-2 inhibition offers several putative advantages. Acting independently of insulin, these agents should not confer a risk of hypoglycemia and could be employed as monotherapy or in combination with other agents. Given their mechanism of action, these agents should be effective in patients with any degree of insulin resistance or β-cell function. They should also be associated with weight loss resulting from the loss of glucose (calories) in urine and glucose-induced osmotic diuresis. Their mild osmotic diuretic effect could potentially also reduce blood pressure. Overall, these effects may have a beneficial impact on cardiovascular outcomes [50].

A delay in reaching HbA1c targets in patients with newly diagnosed T2D is associated with an increased long-term risk of developing CVD, a phenomenon referred to as the legacy effect. The question of whether the early introduction of glucose-lowering drugs with proven cardiovascular benefits, such as SGLT2 inhibitors (SGLT2i), promotes long-lasting benefits in patients who do not achieve proper glycemic control after a type 2 diabetes (T2D) diagnosis remains unresolved [51].

Dapagliflozin, the second FDA-approved molecule following canagliflozin [49], facilitates glucose elimination by the kidneys. Whether used as a monotherapy or in a dual combination with metformin, sulfonylurea, a DPP IV inhibitor, or insulin, dapagliflozin has demonstrated effectiveness in enhancing blood glucose control [52]. Two randomized controlled trials comparing dapagliflozin plus metformin, dapagliflozin alone, and metformin alone showcased significant reductions in HbA1c levels. In Study 1, reductions were −2.05 for dapagliflozin + metformin, −1.19 for dapagliflozin, and −1.35 for metformin (*p* < 0.0001). Similarly, in Study 2, reductions were −1.98 for dapagliflozin + metformin, −1.45 for dapagliflozin, and −1.44 for metformin (*p* < 0.0001). Notably, combination therapy statistically surpassed monotherapy in reducing FPG levels (*p* < 0.0001 for both studies) [53].

Jeon HJ *et al.* (2018) conducted a study to compare a treatment regimen containing basal insulin with two different oral antidiabetic drugs (OAD) (n=148) to a combination drug therapy consisting of dapagliflozin with three other Oral Anti-diabetic Drug (OAD) (n=162) to see which one had a better safety and efficacy profile in T2DM patients with uncontrolled glucose levels. Findings showed that dapagliflozin or insulin glargine both significantly reduced FPG or postprandial 2-hour glucose. After therapy with Dapagliflozin, there was a substantial drop in body weight of -2.36 ± 0.51 kg, but insulin glargine caused an increase of 1.93 ± 0.49 kg (*p* <0.001). The individuals in the dapagliflozin group noticed fewer side effects. These findings imply that the addition of dapagliflozin to an existing medication regimen comprising three distinct OADs in individuals demonstrating insufficient blood glucose control might be other therapeutic strategies in T2DM who hesitate to commence insulin therapy [52].

In 2019, Wiviott, *et al*. conducted a randomized, double-blind, multinational, placebo-controlled, phase 3 trial of dapagliflozin in patients with T2DM and established ASCVD or multiple risk factors for ASCVD (DECLARE-TIMI 58 trial). Seventeen thousand one hundred sixty patients were evaluated, including 10,186 without ASCVD. Major adverse cardiovascular events (MACE) and a composite of cardiovascular mortality or hospitalization for HF were the primary effectiveness outcomes. Death from any cause and a kidney composite were secondary efficacy outcomes. Dapagliflozin, according to the findings, showed positive impacts on several cardiovascular risk variables. Throughout the trial, participants taking Dapagliflozin had lower glycated hemoglobin levels, but it didn't reduce the overall rate of MACE compared to a placebo. Dapagliflozin also significantly lowered the rate of combined cardiovascular death or hospitalization for HF (4.9% *vs.* 5.8%). This benefit was consistent across groups with established heart disease and those with multiple risk factors. While the occurrence of death from any cause was similar between the dapagliflozin group (6.2%) and the placebo group (6.6%), the rate of renal events was slightly higher in the placebo group (5.6%) compared to the dapagliflozin group (4.3%). These findings from the study conclude that in patients with T2DM with or at high ASCVD risk, dapagliflozin lowers the rate of cardiovascular death and reduces the risk of hospitalization for HF and renal outcomes [54].

In prospective, single-arm research, Alguwaihes AM (2021) investigated the safety of dapagliflozin in individuals with T2DM (n=524). In addition, they assessed how HbA1c changed between the baseline and the observation period. The results revealed that the mean (SD) HbA1c % decreased significantly from 8.6 (1.6%) at baseline to 7.4 (1.3%) (*p* <0.0001) after 6 months of treatment with dapagliflozin alone or in combination with other antidiabetic medications. After 12 months of therapy, the mean (SD) HbA1c was found to have decreased even further in comparison to the baseline value, reaching 7.2 (1.2%) (*p* <0.0001). Also, the bulk of unfavorable occurrences were shown to be minor [55].

Similarly, Alhossan *et al.* compared the efficacy and safety of 10 mg dapagliflozin as an add-on therapy to metformin with or without additional OAD in T2DM patients in a retrospective chart review observational trial at King Khalid University Hospital (KKUH) in Riyadh, Saudi Arabia. Two groups of patients participated in this investigation. The dapagliflozin group received metformin and at least one OAD, along with a prescription for dapagliflozin. Those consuming metformin ± 1 or more OAD and not prescribed any SGLT2i made up the control group. Findings showed that between baseline and over the 12-month follow-up period, the mean change in HbA1c for the dapagliflozin group decreased considerably more than that of the control group (-1.22% *vs.* -0.12%; *p* <0.001). As compared to the control group, the dapagliflozin group's change in body weight and body mass index (BMI) was considerably less throughout follow-up from baseline (-1.74 kg *vs.* +0.27 kg; *p* <0.001; and -0.70 kg/m^2^ *vs.* 0.10 kg/m^2^; *p* <0.001, respectively), and these differences were statistically significant. There was also a statistically significant difference in Urinary Tract Infection (UTI) between the dapagliflozin group (10 (12.8%)) and the control group (2 (2.6%)) (*p*=0.032) [56].

In a retrospective chart review study conducted by TawharI, *et al.* at a tertiary care center, all Saudi patients with type 2 DM, aged 18 years or older, who had been taking dapagliflozin for at least 3 months, and who visited the center between August 1, 2021, and July 31, 2022, were included. The results of the present study show that the use of dapagliflozin 10 mg/day in Saudis with DM was safe and resulted in the improvement of glycemic control, lowering of proteinuria levels, and a reduction in the rate of hospitalization. After using dapagliflozin, there was a statistically insignificant increase in the episodes of UTI (12 events *vs.* 7 events). However, there was no episode of ketoacidosis or limb amputation. Further long-term studies with larger sample sizes, preferably randomized controlled studies, are necessary to demonstrate the safety and efficacy of SGLT2 inhibitors in Eastern populations [57].

### 1.15 Adverse Events of Special Interest

Hypoglycaemia occurred in 14% of dapagliflozin and 12% of placebo recipients in the 13-study pooled analysis. Genital infections were more frequent with dapagliflozin than placebo in the 13-study pooled analysis (5.5% *vs.* 0.6%), occurring at least twice as often in women than in men in both treatment groups. All genital infections were of mild or moderate severity, with only 0.2% of patients in the dapagliflozin group and none in the placebo group requiring treatment discontinuation. UTIs were reported in 5% of dapagliflozin and 4% of placebo recipients in this analysis, occurring almost five times more frequently in women than in men, regardless of the treatment group. Treatment with dapagliflozin was associated with small increases in parathyroid hormone, with larger increases seen in patients with higher baseline parathyroid levels. No bone loss was observed with dapagliflozin during 2 years of therapy in patients with normal or mild renal impairment [58].

### 1.16 Cardiovascular Safety

A prespecified meta-analysis of CV events from 21 placebo-/active comparator-controlled phase 2b/3 clinical studies of ≤ 208 weeks’ duration indicated that treatment with dapagliflozin was not associated with an increased CV risk in patients with T2D and suggested a potential CV benefit with treatment, as evidenced by HRs of < 1 for CV outcomes [58].

In conclusion, dapagliflozin was generally well tolerated, with a low risk of hypoglycaemia and drug class-related AEs, including AEs of volume depletion, lower limb amputations, acute kidney injury, and bladder cancer. Diabetic ketoacidosis (DKA) (rare) and genital infections (common), also drug-class related, were reported more frequently with dapagliflozin than placebo; Fournier’s gangrene was reported in one dapagliflozin and five placebo recipients in DECLARE-TIMI [58].

Dapagliflozin exerts its glucose-lowering effects through inhibition of the SGLT2 protein in the kidney proximal tubule, resulting in the excretion of glucose and calories into the urine. This negative energy balance results in dapagliflozin treatment-associated weight loss, as has been demonstrated in several clinical studies. Diabetic patients frequently exhibit a progressive decline in muscle mass and impaired muscle functional quality, which are caused by reduced insulin sensitivity and decreased mitochondrial function due to the underlying pathogenesis of T2DM. Regarding SGLT2i-induced weight reduction, clinical concern has been raised over the occurrence of sarcopenia (decrease in muscle mass), and clinical studies are therefore needed to determine the weight loss efficacy and any effects on muscle mass of dapagliflozin treatment in T2DM patients. Thus Sugiyama S et al, conducted a study to investigate the effects of SGLT2i dapagliflozin treatment on body muscle mass and muscle fat content in patients with T2DM. The study revealed that treatment with dapagliflozin for six months significantly improved glycemic control and reduced body weight without reducing total or skeletal muscle mass in T2DM patients. Regarding the balance between fat and muscle mass, dapagliflozin is promising as a new agent for the treatment of T2DM [59].

Patients receiving SGLT2i consistently experience weight reduction. Meta-analysis had shown that, in comparison to other antidiabetic agents, SGLT2i reduced body weight with a mean difference of 1.8 kg (95% CI: −3.5, −0.1). Moreover, due to these drugs' osmotic diuretic effects, weight loss, may initially appear to be a fluid loss. However, as time goes on, dual-energy X-ray absorptiometry data shows that growing weight loss is most likely the result of caloric loss. Every day, 200-300 calories worth of glucose are eliminated in the urine because of SGLT2i. In a 12-week experiment with dapagliflozin, further weight reduction of 2-3 kg was observed [49].

SGLT2i also reduces systolic blood pressure (SBP) in addition to their antihyperglycemic effects. Kohan DE *et al.* found that dapagliflozin 10 mg dosage lowers SBP and diastolic BP by -6.83 mmHg and -2.53 mmHg at week 1 and by -6.73 mmHg and -2.91 mmHg at week 2, respectively, in a randomized, double-blind, placebo-controlled study on 252 diabetic individuals [60].

### 1.17 Importance of Community Education

Educational level may be the more appropriate, being objective, easy to quantify, and capturing multiple facets of the social determinants of health, including personal behavior, living and working conditions, economic and social opportunities and resources [61]. Peer-to-peer social and emotional support has been shown to help people apply disease management or prevention plans in daily life and links individuals with clinical, community, and other resources. Additionally, studies have shown that the effectiveness of diabetes education on lifestyle modification can be enhanced through the inclusion of peer-to-peer support. Nutrition education is a main component of diabetes education and has been shown to improve dietary behavior and clinical outcomes in persons with diabetes. Moreover, combining the nutrition education program with peer-to-peer support resulted in significantly greater benefits in the reduction of metabolic syndrome in type 2 diabetes [62].

In diabetes management, health education towards self-care empowers patients to make day-to-day decisions about their disease and live a healthy lifestyle. This enables patients to actively participate in the management of their disease based on health education and various skills to achieve targets of metabolic control, prevent or delay the onset of acute and chronic complications, and thus preserve quality of life. This is associated with improvements in self-care behaviors like diet, foot care, physical activity, coping and stress relief, significant clinical outcomes (glycemic control), and acceptability of the condition. There is evidence suggesting that innovative care models pairing community-based support with clinical care may be a more effective strategy for promoting self-management of chronic diseases than clinic or community programs alone, and this is gaining traction among people with diabetes [63].

### 1.18 Panel Discussion

Experts in endocrinology and diabetes management gathered and discussed complications associated with T2DM, patient profiles, treatment goals, ADA 2023 guidelines, and the role of SGLT2i in T2DM management.

According to the experts, before the introduction of insulin in 1922, there was no treatment for diabetes. Insulin in the initial period was obtained from pork and cow pancreases. In the 1970s, DNA technology made it possible to produce human insulin using recombinant DNA techniques. Simultaneously, oral medication such as sulphonylureas and other medications were developed for diabetes management. Experts report that approximately 90% of diabetes cases worldwide are T2DM. The remaining 10% includes T1DM, MODY, GDM, and other types, such as those induced by pancreatic issues or medications. In KSA, the majority of diabetes seen among the Saudi population was T2DM. The prevalence is still increasing. Several studies on the prevalence of diabetes have been published in KSA. The Saudi-DM research by Al-Rubeaan was the most detailed. According to the study, abnormal glucose metabolism has reached epidemic proportions in the KSA, with more than 50% of the Saudi population aged ≥30 years being either diabetic (25%) or pre-diabetic (25%), and an alarming 40% of diabetic patients were unaware of their disease [29]. Another study revealed that the prevalence of diabetes in KSA is 16%. In this study, 10,000 individuals were screened for 5 years. This was a randomized study conducted in all regions of KSA. However, the problem with this study was that younger patients (18 years of age) were enrolled, and they used HbA1c for monitoring rather than fasting or postprandial glucose. Younger patients have less chance of getting diabetes. IDF reported in its recent data that the total diabetes prevalence in KSA is 17%, whereas, in its previous edition, it was 20% [7]. These data do not necessarily indicate that the prevalence of diabetes has decreased in KSA, as many factors may affect the prevalence such as the region where the study was conducted, age distribution, disease duration, *etc.*

In addition, researchers have also shown that the prevalence of pre-diabetes is considerably greater in KSA. If one includes pre-diabetes statistics, the prevalence of the disease will rise even more in KSA.

According to KSA demographic data, the Jizan region has the lowest prevalence of diabetes, while the northern region has the highest. The Saudi National Diabetes Center is carrying out a project/investigation program in the nation. The center has thus far gathered screening data on 800,000 people from the north and south areas. These data revealed that 40-50% of patients have diabetes but are unaware of it. The center is currently aiming to extend the screening program to institutions so that those centers may gather their data. It helps to have an in-depth look at the complications of diabetes (neuropathy, nephropathy, retinopathy, *etc.*). Mokdad *et al.* (2015) revealed that the economic burden of diabetes in KSA for all Saudis was around 17 billion riyals, and it is suggested that the current cost may have risen to 20 billion riyals [33].

Obesity is a risk factor for diabetes. In KSA, the frequency of obesity is also rising (affecting 40-50% of the KSA population). Diabetes risk is increased by 30-40% in obese adults over 30 with a BMI of 30 or higher. Recent obesity statistics showed that most obese patients lived in the West, followed by the Central region. A survey was performed by Akshar at the time of COVID-19 (not yet published). The survey asked questions on diabetes and other risk factors. The figures obtained from this survey were similar when compared to the figures of the Ministry of Health (MoH). 70-80% of the participants in this survey were male, and 20% were female. According to IDF research, if the KSA population does not change their lifestyle habits, 40-50% of people will get diabetes by 2030. These stats suggest that by 2040 or 2050, 20% of the KSA population will be disabled by diabetic neuropathy, nephropathy, and retinopathy. T2DM cases in children and young people in KSA have recently been rising. T2DM is typically not diagnosed in youngsters between the ages of 13 and 14. Perhaps an elevated risk of obesity is the root cause of T2DM.

Besides social support, building future communities that include fitness centers, parks, cycling lanes, and sidewalks is needed to promote healthy lifestyles among the Saudi population. Policies to increase access to grocery stores and limit access to fast food could lead Saudis to have better diets, lower obesity rates, and potentially reduce the risk of T2D. Indeed, cooperation between public health providers, clinicians, community leaders, gatekeepers, and stakeholders would be useful in identifying the needs of each community’s access to healthy food and recreation centers. These partnerships may lead to effective diabetes prevention and management in each Saudi community [64].

Experts highlighted that Saudi Arabia has no clear or standardized patient journey both in private and government settings, the journey differs. Patients usually go to the GP for medication, and if their symptoms are not controlled by GP medication, they may go to the diagnostic center’s major hospitals to get a proper diagnosis of the disease. However, there is a big gap/lag when it comes to diabetes prevention and complications. At present, the most common cause of renal disease, amputations, retinopathy, cardiovascular death, *etc.*, is diabetes.

Screening and prevention of diabetes and its complications are primary. However, even in good facilities, risk factors are not always handled and are occasionally disregarded. At certain facilities, 50% of patients are not even screened using HbA1c. Worldwide statistics show that 50% of doctors do not calculate eGFR. Similarly, 30-40% of specialist doctors did not calculate the albumin-creatinine ratio. Other factors also play an important role when it comes to managing diabetes — some hospitals do not recommend combined medication and/or polypharmacy (5 or more drugs). All of these are serious issues that need to be fixed. Advances in this area will enhance patient care, quality of care, and complications. King Fahd Medical City is one of the hospitals that provide combined medicine.

In urban and rural regions, diabetes prevalence might differ. Diabetes is more prevalent in urban locations in certain nations, whereas it may be more prevalent in rural regions in other nations. According to studies, 25% of undiagnosed diabetes cases occur in high-income households, whereas 50% of cases are found in low- or middle-class families. The low diagnosed instances in higher-class households may be attributable to glucometer accessibility or availability, whereas the high undiagnosed cases in rural regions may be related to low education.

Knowing the importance of investigation can influence treatment decisions. Everyone in the private sector can visit any major consultant for disease investigation and management, regardless of insurance status. Every institution has a primary care center, where patients often go when they need medical attention. The patient is sent to a tertiary care facility if the primary care facility is unable to handle the patient's ailment. Most institutions, including the National Guard and the Ministry of Defense, have primary or tertiary care facilities. However, they lacked secondary healthcare. All three levels of care—primary, secondary, and tertiary—are offered by the MoH. Patients in the MoH often go through primary care first, then, if necessary, are moved to tertiary care.

Experts emphasized that HbA1c levels should be monitored every three months for most patients and every two months for those with uncontrolled diabetes. In gestational diabetes, routine HbA1c testing is advised. The Ambulatory Glucose Profile (AGB) test is also one of the most important determinants of good glycemic management. It can differentiate between good and bad glucose levels, which helps doctors/physicians manage diabetic patients. If the AGB report is checked with Continuous Glucose Monitoring (CGM) it will help determine many cases of hypo- and hyperglycemia. However, the issue is that most patients and doctors think CGM is not necessary. Glycemic variability could be a crucial sign of how well diabetes is controlled. It could be acute or chronic. The only way to determine glycemic variability in T2DM is through AGB reports, and weight loss and weight control are the best ways to control it. However, with T2DM, patient motivation for weight control is most important. According to an MoH study on self-glucose monitoring, those who consistently monitor their blood sugar have better glucose management than those who don not check it often (patients will continue taking medicine).

Treating T2DM will help prevent complications. Discussing T2DM-related complications, experts revealed that 12% of patients in the pre-diabetic stage may suffer from retinopathy, 11% may suffer from neuropathy, and some have other diabetes-related risk factors. Sometimes complications develop before diagnosis, while other times, the beta-cell function starts to decline before the diagnostic stage. When observing T2DM patients, physicians often notice that the problem could have begun 4-5 years earlier.

Screening for complications of diabetes is extremely important. All guidelines state that once T2DM is diagnosed, the patient is immediately screened for all risk factors. However, access to different specialties is the problem. The solution to this problem is to conduct a detailed, comprehensive examination every time patients visit the doctors (diabetologists, endocrinologists, ophthalmologists, and dietitians for nutrition recommendations). All patients are evaluated for HbA1c, lipid profile, blood pressure, albumin-creatine ratio, proteinuria, and fundoscopic examination, including those with cardiovascular risk factors, smoking, stroke, *etc.* These tests should be repeated at least once a year. If the results of these tests are abnormal or individuals begin to show symptoms, testing may be done more frequently, such as every 6 months, every 3 months, or every 6 weeks, as per the individual’s requirement. When the optical coherence tomography (OCT) program was launched in KSA, over 10,000 individuals were waiting to be examined by ophthalmologists, however, at present, almost all patients have been checked.

Experts discussed patient profiles and the goal of T2DM, and they revealed that patient profiles are not the same for all patients. Patients who visit the doctor usually are not the same because diabetes is a very complicated disease that requires therapy that matches its complexity. In adults ≥18 years old. HbA1c is more flexible (up to 8) because of comorbid illnesses like ischemic heart disease. If the patient has cancer or is asymptomatic, then their HbA1c may even be higher (up to 8.5 or 9). Thus, handling the A1C will help in avoiding complications like hypoglycemia and others.

A patient-centered (individual patients' specific symptoms and preferences were assessed to develop an individualized treatment plan and therapy objectives) and weight-centered approach (set individualized weight management goals) is used in T2DM management. Increased patient satisfaction, enhanced patient-provider communication, improved outcomes, enhanced patient well-being, and improvement in QoL are all advantages of these two approaches. Before this, there was a pro-concentric approach (focusing on HbA1c readings only) and a cardio-centric approach (SGLT2i or GLP-1RA are added to prevent heart disease).

Nowadays, doctors' approach most of their patients with a weight-centric approach. In this approach, physicians obtain a thorough history of the patient’s lifestyle, nutrition, and physical health. They also make changes to his/her medication, removing sulphonylurea and insulin and replacing them with SGLT2i and GLP-1RA, which have proven cardiovascular and renal benefits. However, this approach differs from person to person. A healthy lifestyle, which includes exercise, daily walking for at least 30 minutes, and a lifestyle change, is mostly individualized.

Clinical inertia is also a barrier to treatment effectiveness in the management of diabetes and its consequences. This causes management to be delayed (sometimes for more than 2-3 years). Three groups of factors may cause clinical inertia: those pertaining to patients, healthcare providers, and the healthcare system. Doctors need to emphasize overcoming this barrier. Additionally, doctors should manage their patients in a way that the drugs used do not lead to hypoglycemia.

Experts noted that some doctors and physicians do not stay updated with current diabetes treatment guidelines, which can lead to inadequate diabetes care. A major problem in KSA is the health system and the roles of physicians and patients. Doctors always blame patients for not controlling diabetes, but if we look at the literature, 50% of patients are uncontrolled due to misunderstanding by doctors, and 20-25% may be a healthcare-related issue. In 25% of cases, the patient is also responsible. However, the biggest part belongs to the doctor. Perhaps sometimes, the lack of clear guidance, poor access to care, and problems with patient-doctor communication obscure the way to manage diabetes. Pharmaceutical companies can help with this problem by providing people with training, supervision, case-based discussions, and communication. This will help people to fly again (*i.e.,* feel confident). Sometimes, people are scared, and they do not use proper management protocols. They can also fly solo if they use medication under supervision or are trained or educated.

Before 2021, one of the gaps in managing diabetes in the KSA was the lack of national diabetic guidelines. Clinicians had to adhere to the ADA, Canadian, or Australian guidelines, which were widely available. However, these guidelines were challenging to read, as they comprised 200-300 pages. Moreover, they did not reflect the local data and needs of the KSA diabetic population. In 2021, the Saudi National Diabetes Center (SNDC) developed the Saudi Diabetes Clinical Practice Guidelines (SDCPG) for Saudi KSA physicians, based on the data from the international guidelines and studies published on the KSA diabetic populations. The SDCPG guidelines have 12 chapters, covering various aspects of diabetes management, such as screening, prevention, assessment, goal setting, treatment plan, lifestyle modification, pharmacologic approaches, blood glucose monitoring, managing cardiovascular risks, managing acute and chronic diabetic complications, and managing special populations. These guidelines provide detailed information for managing KSA diabetic patients, reflecting the local demographic data and needs.

After being accepted, the KSA pharmaceutical industry promoted these guidelines as national recommendations endorsed by the Saudi Health Council for KSA diabetic populations. Additional strategies for dissemination include encouraging hospitals to post them on websites and build consensus around the guidelines.

These guidelines helped raise awareness that there was local content for local people that reflected local demographic data and needs. The SDCPG guidelines aimed to improve the quality of care and outcomes for KSA diabetic patients [1].

Experts discussing the role of SGLT2 inhibitors, specifically dapagliflozin, highlighted the influence of ADA (2022) and SDCPG (2021) guidelines in facilitating drug selection based on patient conditions. The current guidelines favor SGLT2 inhibitors as the primary choice for managing T2DM, a notable shift from a few years ago when sulfonylurea held that position. SGLT2 inhibitors, such as dapagliflozin, contribute to weight reduction and demonstrate efficacy in reducing cardiovascular adverse outcomes in individuals with T2DM. As per the ADA (2022) and SDCPG (2021) guidelines, SGLT2 inhibitors, particularly those with cardiovascular benefits, can be considered for individuals with cardiovascular disease (CVD) and uncontrolled glycemia on existing medications, provided they have an estimated glomerular filtration rate (eGFR) greater than 30 mL/min/1.73m^2^. Additionally, SGLT2 inhibitors are recommended as a first-line therapeutic option for non-diabetic individuals, particularly those with HF. For patients at an increased risk of ASCVD, HF, or CKD, SGLT2 inhibitors are preferred as a second-line therapy, taking into account drug-specific and patient factors. The potential benefits of SGLT2 inhibitors extend to dialysis patients, although specific guidelines for this population are currently unavailable [1].

If a patient has weight problems, GLP-1RA and SGLT2i may promote weight loss. DPP4, metformin, GLP-1RA, and SGLT2i drugs minimize the risk of hypoglycemia. The ADA guidelines also recommend SGLT2I as one of the drugs for congestive HF patients (HFrEF or HFpEF). It reduces the chances of cardiovascular mortality and hospitalization. Concerning CKD, the ADA divided patients into 2 groups: one group had progression of CKD, while the other had microalbumin urea. The guideline advocates starting an SGLT2i for patients who have an eGFR ≥20 ml/min per 1.73 m^2^. GLP-1RA with proven CVD benefit if not tolerated or antagonized by SGLT2i. GLP-1RA usage in ASCVD is restricted in KSA, both in government and private institutions, due to misuse. However, there are no limitations on the use of SGLT2i in ASCVD in either private or government hospitals. Patients with an eGFR below 20 ml/min per 1.73m^2^ will not benefit from SGLT2i.

Cardiovascular evaluation in diabetes was started after the rosiglitazone warning. One study showed that rosiglitazone is associated with a higher risk of HF in individuals with T2DM. When treating HbA1c, cardiorenal safety is also considered. Physicians can run across issues like whether to start SGLT2i in patients who are already on insulin or MDIs or whether there is a risk of hypoglycemia. Given the complexities of managing diabetes, some clinicians often discontinue prescribing weight-gain medications and begin prescribing SGLT2i and GLP-1RA if they are available. This is the limitation of certain guidelines.

“Modification” is another issue that physicians encounter when managing diabetes. There are no guidelines that discuss how to replace the present drug with some other medicine in clinical practice. This approach is not complicated, though. Add-on medicines are not an issue for individuals with uncontrolled diabetes with HbA1c ≥9 since there is no risk of hypoglycemia. Nevertheless, before adding another medicine to the therapy, the patient profile is considered. The treatment's cost is also a concern. Some do not have health insurance and cannot afford expensive medications.

According to experts, SGLT2i is one of the drugs that has high efficacy (glycemic control) and is better than sulfonylurea, DPP4i, and other drugs, and in the pursuit of weight loss, it is one of the intermediate drugs.

Furthermore, pharmaceutical companies have launched diabetes screening initiatives in the MENA region, according to experts. This can assist people in being aware of and informed about the illness, as well as receiving treatment at the right time.

## CONCLUSION

T2DM incidence appears to be increasing in the Saudi population, creating a serious public health concern in the country. Thus, it is essential to develop enhanced HRQoL for persons with diabetes to reduce societal and personal costs associated with diabetes treatment in KSA. For the treatment of T2DM, the SGLT2i family of drugs offers an effective and typically well-tolerated alternative.

These treatments can help patients achieve and maintain blood glucose control. Each patient profile, however, is unique. The same medication cannot be administered to several patients. Choosing the right medication for each patient requires caution. The cost of the medication was also considered when choosing the drugs.

## Figures and Tables

**Fig. (2):**
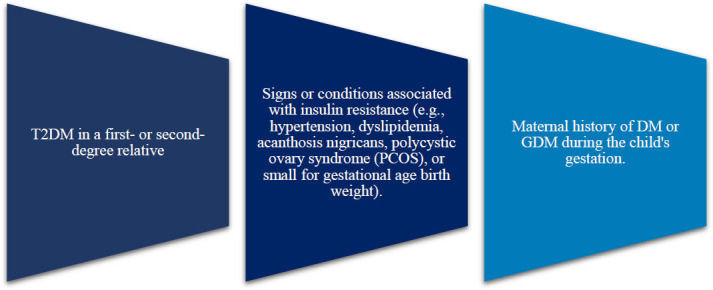
Screening of T2DM in asymptomatic adolescents.

**Table 1:** Diagnosis of DM and prediabetes.

-	**Fasting Blood Glucose (FBG)**	**2-hours Postprandial Blood Glucose (PPBG)**	**Glycosylated Hemoglobin A1c (HbA1c)**	**Random Blood Sugar (RBG)**
**Prediabetes**	>100 mg/dl but <126 mg/dl (>5.6 but <7 mmol/L)	>140 mg/dl but <200 mg/dl (>7.8 but <11.1 mmol/L) during 75 gm OGTT	>5.7 to 6.4%	-
**DM**	>126 mg/dl (7 mmol/L)	>200 mg/dl (11.1 mmol/L) during OGTT	>6.5%	>200 mg/dl (11.1 mmol/L)

## LIST OF ABBREVIATIONS

SGLT2i: Sodium-Glucose Transport Protein 2 Inhibitors
T2DM: Type 2 Diabetes Mellitus
DM: Diabetes Mellitus
KSA: Kingdom of Saudi Arabia
WHO: World Health Organization
